# Evaluation of a quality improvement intervention for obstetric and neonatal care in selected public health facilities across six states of India

**DOI:** 10.1186/s12884-017-1318-4

**Published:** 2017-05-02

**Authors:** Enisha Sarin, Subir K. Kole, Rachana Patel, Ankur Sooden, Sanchit Kharwal, Rashmi Singh, Mirwais Rahimzai, Nigel Livesley

**Affiliations:** 1 Consultant, University Research Company, India Pvt. Ltd B7, 1st floor, Suncity, Gurgaon, 122002 Haryana India; 2 Data Manager, University Research Company, India Pvt. Ltd, T8-502 Amrapali Grand Sector Zeta 1, Greater Noida, 201306 UP India; 3 Consultant, University Research Company, India Pvt. Ltd. E 5, NTRO scientist Hostel, Behind Sahastra Seema Bal, Aya Nagar, Delhi 110047 India; 4 Senior Advisor, University Research Company, 1st floor, LMR House, S-16, Uphaar Commercial Complex, Green Park Extension, New Delhi, 110016 India; 5 Doctoral Fellow (Social Epidemiology), Humanities and Social Sciences Discipline, Indian Institute of Technology, Gandhinagar, India; 6 Lead- Quality and Process Improvement, ACCESS Health International, Nilgiri building, IIIT, Gachibowli, Hyderabad, India; 7 Project Director, University Research Company, Plot 40, Ntinda II Road, Kampala, Uganda; 8 Project Director, University Research Company, 1st floor, LMR House, S-16, Uphaar Commercial Complex, Green Park Extension, New Delhi, 110016 India

**Keywords:** Quality improvement, Obstetric care, Neonatal care, Health care delivery, Health system strengthening, India

## Abstract

**Background:**

While increase in the number of women delivering in health facilities has been rapid, the quality of obstetric and neonatal care continues to be poor in India, contributing to high maternal and neonatal mortality.

**Methods:**

The USAID ASSIST Project supported health workers in 125 public health facilities (delivering approximately 180,000 babies per year) across six states to use quality improvement (QI) approaches to provide better care to women and babies before, during and immediately after delivery. As part of this intervention, each month, health workers recorded data related to nine elements of routine care alongside data on perinatal mortality. We aggregated facility level data and conducted segmented regression to analyse the effect of the intervention over time.

**Results:**

Care improved to 90–99% significantly (*p* < 0.001) for eight of the nine process elements. A significant (*p* < 0.001) positive change of 30–70% points was observed during post intervention for all the indicators and 3–17% points month-to-month progress shown from the segmented results. Perinatal mortality declined from 26.7 to 22.9 deaths/1000 live births (*p* < 0.01) over time, however, it is not clear that the intervention had any significant effect on it.

**Conclusion:**

These results demonstrate the effectiveness of QI approaches in improving provision of routine care, yet these approaches are underused in the Indian health system. We discuss the implications of this for policy makers.

**Electronic supplementary material:**

The online version of this article (doi:10.1186/s12884-017-1318-4) contains supplementary material, which is available to authorized users.

## Background

Since the launch of the conditional cash transfer programme, Janani Suraksha Yojana (JSY), the percentage of women delivering in institutions in India increased from 56.7% in 2006 to 78.5% in 2011 [1]. However, maternal mortality and neonatal mortality did not decrease commensurately. An analysis of annual health survey data from nine states found no association between institutional birth proportion and maternal mortality. The authors suggested that this could be due to poor quality of services [2]. Other studies have found evidence to support the contention that poor quality of care explains why mortality remains high, reporting lack of routine care, absence of organized preparation to conduct birth, staff abuse and neglect of women during delivery [3], lack of skilled birth attendants, referrals that did not result in treatment [4], and low competence among staff to manage obstetric complications [4, 5].

There are also concerns with quality of care for neonates and infants. Available evidence from facilities in India and other South Asian settings point to low rates of asphyxia management [6] and low coverage of immediate breastfeeding and thermal care [7].

These data support the premise that poor quality of care is a major contributor to maternal and newborn mortality in India. Therefore, there is an urgent need for approaches which can support providers to deliver better care. A recent publication lists various strategies to improve service delivery [8]. These include development of standards and guidelines, changing organizational structures, education and training, process improvement, providing incentives, changing organizational culture, and leadership or management interventions [8].

The Indian public health system uses most but not all of these approaches to improve maternal and neonatal health. Standards and guidelines exist [9]; new organization structures such as the national RMNCH + A unit (NRU) and State RMNCH + A units (SRU) have been formed to focus on maternal and neonatal health [10]; there are extensive in-service training programs; incentives, campaigns and institutional frameworks include quality issues [11]; and many inspection/monitoring systems exist. Despite the use of this diverse set of interventions, process or quality improvement methods are underused.

Quality improvement (QI) is a management approach that equips health workers with the skills that allow them to change how they work to ensure that they can apply evidence-based practice [12]. QI is based on the principles of focusing on the client’s outcomes, thinking in terms of systems and processes, teamwork, using data to guide decision making, and iterative testing to change service delivery [13].

Between January 2014 and November 2015, the USAID Applying Science to Strengthen and Improve Systems (ASSIST) Project worked with 125 public health facilities across six states in India which deliver approximately 180,000 babies per year. The project supported health care staff to use QI approaches to redesign processes and ensure that appropriate care was delivered. In this paper, we report on the efforts to improve elements of routine care using QI approaches.

## Methods

### Intervention sites

In collaboration with the Ministry of Health and Family Welfare, we supported 27 high priority districts in six states - Delhi, Haryana, Himachal Pradesh, Jharkhand, Punjab, and Uttarakhand. Hence, the selection of intervention sites are purposive in nature. MoH had identified these districts based on selected key performance indicators in RMNCHA domain. These comprised the bottom quintile of all districts in each state in terms of performance and also took into account equity concerns. Within these districts, we planned to have an impact on maximum number of deliveries that was possible for one coach in every district to handle. In each district, we worked at the district hospital, sub district hospital/s, and all primary and community health centres in a block selected on the following criteria: 1) willingness of block leaders to implement a QI project, 2) accessibility, and 3) mid-level performance^1^ on health indicators. This site selection allowed us to work for 50–80% of child-births occurring in government health facilities in every district. In these facilities, care is provided by nurses, doctors and specialists (OBGYN and Pediatrics) who composed a QI team. Whenever required and available, additional staff such as pharmacist, helpers, ASHAs (community health workers), facility head, and auxiliary nurses were involved.

### Description of the QI intervention

After identification of facilities, project staff provided initial training to health workers and managers on the seven steps of quality improvement, laid down in a QI Implementation guide [14]. This included an introduction to how to: 1) identify an improvement goal, 2) form and work in a team, 3) analyze current systems of care to identify the reasons for poor care, 4) develop simple measurement systems, 5) develop options for possible solutions, 6) test these interventions on a small scale, 7) implement the interventions that were found to be successful. Interventions covered systemic and process changes including facility improvement, supplies of medicines, improved record keeping and so on depending on the nature of the problem identified.

After the initial training, project staff visited facilities roughly every month to provide hands-on support to health workers in applying these skills to identify and solve problems of patient care. The project employed one coach per district to provide this ongoing support. Initially, facility staff were encouraged to choose improvement goals involving routine care (preventative, diagnostic, or management interventions) that all pregnant and post-partum women and all neonates should receive. In this paper, we look at the effect of the QI support on nine elements of routine care and perinatal mortality.

### Data collection

Data were self-collected by the teams of health workers and came from existing hospital records which included patient case files, ANC cards and delivery registers. These data are routinely collected as part of the ANC, delivery and post partum procedures, and we did not collect additional data. Each month, project staff verified and recorded data in a predesigned Excel database. Data were then aggregated at district, state, and finally at aggregate level, with data verification at each step. Additional files are attached to Additional file 1 indicate proportion of services (indicators) conducted, and Additional file 2 to show aggregate number of patient records reviewed for ANC, delivery and newborn care across all months. Because facilities only reported data related to the goals that they were working on, the number of facilities reporting data for each indicator varies. Data reporting started as soon as district coaches were hired and QI teams formed, from July 2013 onwards. Most of the indicators were taken up for improvement following the QI model, from January to February 2014. Data collection continued till August 2015 when the project officially closed.

### Ethical clearance

The data pertain to an intervention project and are taken from hospital records that are routinely collected and hence no ethical clearance was required. This was a basic program evaluation that was a routine part of implementation with no additional data collected than would be normally expected in such a program. Anonymity and patients’/clinicians’ rights were respected and we had permission from facilities plus MOH to implement the intervention. Furthermore, as per the national guidelines for biomedical research, “research on publicly available information, documents, records, works, performances, reviews, quality assurance studies, archival materials or thirdparty interviews, service programs for benefit of public having a bearing on public health programs, and consumer acceptance studies” are waived from voluntary informed consent process [15]. The use of data for a quality improvement intervention falls in the above mentioned category.

### Measures

For tracking the progress of our intervention, we selected a set of nine process indicators spread across four domains of antenatal (ANC), intrapartum, essential newborn care (ENC), and postpartum care. These indicators were chosen based on prior experience of the implementers about which elements of care are good to teaching health workers how to use QI approaches. Facility staff chose new indicators to work on as their skills grew based on what they perceived to be priorities in their facilities, which are not presented in this analysis as the start time differs. The current analysis focuses on the nine process indicators that the QI teams worked on at the start of the USAID ASSIST support. Additionally, we included perinatal mortality indicator to see the effect of the intervention. The specific indicators were:

ANC:

1. Proportion of antenatal care visits (ANCs) during which the hemoglobin of pregnant woman was documented;
2. Proportion of ANCs during which history was taken to rule out high-risk pregnancy;
3. Proportion of ANCs during which counseling about nutrition, family planning, breastfeeding was documented.

Intrapartum:

4. Proportion of vaginal deliveries for which a uterotonic was administered within one minute of birth of baby.

Essential newborn care:

5. Proportion of newborns made dry and provided warmth immediately after birth;
6. Proportion of newborns provided sterile cutting and clamping of cord;
7. Proportion of newborns breastfed within one hour of birth;
8. Proportion of newborns given injection of vitamin K within 24 h of birth.

Postpartum Care:

9. Average number of times blood pressure and pulse rate were recorded within the first six hours after delivery.

Outcome Measure:

10. Perinatal mortality rate calculated by totaling neonatal deaths and still births over the total number of live births in a facility.

### Data analysis

#### Trend analysis

Most facilities started their QI projects in January– February 2014, with baseline data collected for the preceding months. Therefore, we have month 8 (February 2014) as the cutoff point preceding which is baseline data and following which is intervention data. Table 1 shows the number of facilities reporting on selected indicators, which varies as new facilities get added up or older facilities may stop reporting once they have achieved their goal.

**Table 1:** Number of facilities reporting data

Number of facilities covered									
Months	Hb check	History taken	ANC counseling	Keep new born dry and warm	Sterile Cord care	Vitamin K	Early Breast feeding	Oxytocin given	Postnatal vitals check
Jul-13	50	41	39	58	59	83	72	101	74
Aug-13	52	43	40	58	59	83	72	101	74
Sep-13	52	43	40	58	59	83	72	101	74
Oct-13	53	44	41	58	59	83	72	101	74
Nov-13	54	44	41	58	59	83	72	101	74
Dec-13	56	46	44	58	59	83	72	101	74
Jan-14	69	54	53	58	59	83	72	101	74
Feb-14	73	56	53	62	63	89	82	102	84
Mar-14	73	55	53	74	75	99	86	111	88
Apr-14	71	48	46	70	70	100	86	115	87
May-14	58	36	34	58	59	102	74	116	78
Jun-14	57	35	33	57	58	109	74	117	87
Jul-14	44	22	20	53	54	109	77	116	88
Aug-14	46	24	22	54	55	109	81	118	91
Sep-14	46	24	22	53	54	107	86	118	90
Oct-14	49	26	24	52	53	107	89	117	90
Nov-14	51	28	26	51	52	107	90	117	90
Dec-14	51	28	26	51	52	108	90	118	91
Jan-15	49	26	24	51	52	102	84	111	91
Feb-15	49	26	24	51	52	102	84	111	91
Mar-15	50	27	25	51	52	108	90	117	91
Apr-15	49	26	24	51	52	108	90	117	91
May-15	45	26	24	51	52	108	89	117	90
Jun-15	45	26	24	51	52	108	89	117	90
Jul-15	40	26	24	49	50	103	84	109	85
Aug-15	40	26	24	49	50	101	82	107	84

Firstly, to explore time trend from July 2013 to August 2015, we aggregated data from all states at the national level for selected indicators, calculated proportions, and plotted them on graphs.

Secondly, facility-wise data were aggregated at state level for the entire time period (26 time points) and then transferred to Stata version 13.1 for segmented regression analysis. This analysis was conducted to see the effect of the intervention on service delivery at various points of the project life.

#### Estimating changes in level and trend: segmented regression

To assess chance and control for other effects, segmented regression analysis was used. Each segment of the series was allowed to exhibit both a level and a trend that followed an intervention. This statistical model estimates level and trend in the pre-intervention segment and changes in level and trend after the intervention.

1 $$ {Y}_t={\beta}_0\kern0.5em +{\beta}_1\ast \kern0.5em  t i m{e}_t+{\beta}_2\ast interventio{n}_t+{\beta}_3\ast time\; after\kern0.5em  i nterventio{n}_0+{\varepsilon}_t $$

Here, Y_t_ is the proportion of women obtained services for selected health indicator under QI measures in month t; *time* is a continuous variable at time t from the start month of the observation period; *intervention* is an indicator for time t occurring before (intervention = 0) or after (intervention =1) the cap, which was implemented at month 8 in the series; and *time after intervention* is a continuous variable counting the number of months after the intervention at time t, coded 0 before the cap and (time—9) after the cap.

In this model, *β*_0_ estimates the baseline level of the outcome at time 0; *β*_1_ estimates the change in the proportion that occurs with each month during the intervention; *β*_2_ estimates the level change in the proportion immediately after the intervention, that is, from the end of the preceding segment; and *β*_3_ estimates the change in the trend in the proportion after the cap. The error term *e*_*t*_ at time *t* normally distributed random error that may be correlated to errors at preceding or subsequent time points [16–18].

## Results

We present the results in trend charts which uses data aggregated from all six states at the national level. Furthermore, we present the segmented regression analysis which uses data from all facilities at the state level. Overall, bivariate analysis shows that the process indicators improved significantly post intervention compared to pre intervention (Table 2).

**Table 2:** Test of means of measures by intervention group

Measures	Prior to intervention	During intervention	Aggregate	*p* (ttest)
Hemoglobin checked	0.53	0.87	0.77	0.0000
ANC history taken	0.12	0.77	0.58	0.0000
ANC counseling	0.05	0.71	0.50	0.0000
Oxytocin within 1 min	0.19	0.95	0.74	0.0000
Kept baby DRY & warm	0.36	0.98	0.83	0.0000
Sterile cord care	0.36	0.97	0.84	0.0000
Early breastfeeding	0.35	0.95	0.79	0.0000
Vitamin K	0.76	0.90	0.87	0.0000
Average no. of times PNC	0.69	3.01	2.35	0.0000
Perinatal Death Rate (per1000 live births)	26.73	22.92	24.09	0.0084

### Antenatal care practices

**Fig. 1:**
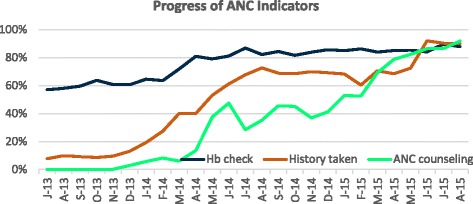
Trend in ANC indicators over baseline and intervention period (July 2013– August 2015)

Performance of desired antenatal care practices increased considerably over the intervention period (Fig. 1). Segmented regression results (Table 3) indicate that just before the beginning of the observation period, on average 40% (adjusted for time and intervention) of women went for Hb test. With time, there was significant month-to- month change of 0.04 in the proportion of Hb test (*P* < 0.014)) and even right after the cap, the estimated proportion increased abruptly by 0.17. There was no significant change in the month-to-month trend in the mean number of tests after the cap, as the trend of approximately 85% was maintained across the months of intervention. Proportion of ANC history taking increased by 0.30 over time with the intervention having a significant impact (*P* < 0.001). Similarly, ANC counseling significantly increased over time as well as during the time after intervention by 0.025 (*P* < 0.001).

**Table 3:** Full segmented regression model

Health Measures	Intercept	Time	Intervention	Time of Intervention
	*β*_0_ (SE)	*β*_1_ (SE)	*β*_2_ (SE)	*β*_3_ (SE)
Hemoglobin checked	0.36 (0.071)	0.036**(0.014)	0.175*(0.071)	−0.031 (0.014)
ANC history taken	−0.010 (0.074)	0.029*(0.014)	0.297***(0.074)	−0.002 (0.015)
ANC counseling	0.047 (0.053)	0.024 (0.023)	0.439***(0.090)	0.025***(0.007)
Oxytocin within 1 min	−0.070 (0.049)	0.056***(0.009)	0.495***(0.048)	−0.047***(0.010)
Kept baby DRY & warm	−0.353 (0.055)	0.131***(0.009)	0.259***(0.040)	−0.129***(0.010)
Sterile cord care	−0.346 (0.055)	0.130***(0.009)	0.242***(0.040)	−0.127***(0.010)
Early breastfeeding	−0.049	0.081***	0.281***	−0.074***
Vitamin K	0.940 (0.065)	−0.035**(0.012)	0.202***(0.053)	0.039**(0.012)
Average no. PNC	0.107 (0.413)	0.125 (0.079)	0.747*(0.401)	−0.002 (0.082)
Perinatal Death Rate (per1000 live births)	31.77***(2.853)	−1.118**(0.565)	2.818 (2.971)	0..832 (.588)

*p < 0.05, **p < 0.01, ***p < 0.001

### Intrapartum care

**Fig. 2:**
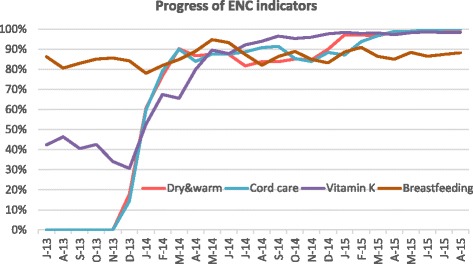
Trend in Essential Newborn Care indicators over baseline and intervention period (July 2013– August 2015)

**Fig. 3:**
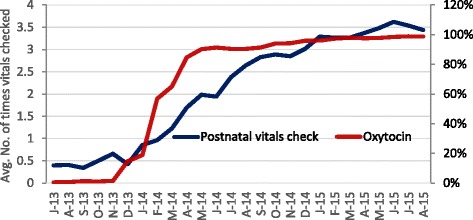
Trend in Oxytocin administration and maternal vitals monitoring over baseline and intervention period (July 2013– August 2015)

**Fig. 4:**
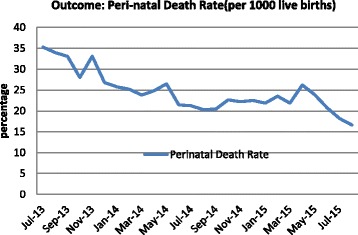
Trend in perinatal mortality and post- partum hemorrhage over baseline and intervention period (July 2013– August 2015)

Similar results to antenatal practices regression model explains the proportion of women given oxytocin within one minute of delivery, proportion of newborns kept dry & warm, sterile cord care, early breastfeeding and vitamin K provided were significantly (*p* < 0.001) increased by 20–70% during post intervention period and month-to-month significant (*p* < 0.001) improvement (3–13%) was also observed (Table 3). As can be seen from the time series chart (Figs. 2, 3 and 4), the indicators began to increase during the preparatory phase of the intervention while after February 2014, except Vitamin K, the provision of all other practices stabilized at around 90% during the early months of intervention (breastfeeding at around 85%). Vitamin K provision, however, kept increasing significantly from month to month during the period of intervention (*p* < 0.01). It took 8–9 months to increase vitamin K injection from 40 to 70%, after which it was sustained at 98% until the end of the project.

### Postpartum care

Postpartum care in terms of checking of mother’s vitals also improved over the intervention. The average number of times that blood pressure and pulse was checked for a woman within 6 h after delivery increased from 0.3 to 3.5 times (Fig. 3). Segmented regression analysis shows that while post intervention had a significant impact in its increase (75%, *p* < 0.05), there was no significant increase in the month to month trend during the period of intervention.

While perinatal mortality decreased as a function of time (*P* < 0.01), the pre-post intervention showed no significant impact, nor did the trend show any month to month change during the period of intervention (Table 3).

In general results from segmented model confirms month-to-month and pre-post intervention period significant improvement in health care indicators whereas trend after intervention did not have the same effect in variation (barring some indicators) which is probably due to a rapid and huge increase in the early phase of intervention following which many of the indicators stabilized at a constant rate.

## Discussion

There is growing interest in how to improve quality of care for maternal and newborn health in developing countries [19]. Most literature that exists focuses on single interventions such as training, audits, guidelines, or improving facility infrastructure [20–24], and ignores the growing body of evidence that draws from scientific management approaches, particularly those developed in the auto industry, which performs very well on quality measures [25, 26].

This paper addresses this gap in the literature by describing how a QI approach led to improved processes of care across a wide range of clinical areas and wide range of clinical contexts (rural, urban, large, small, etc.). Health workers in selected facilities in 27 districts with a combined population of 32 million people were supported to use QI approaches by externally funded QI coaches (1 per district). This approach rapidly led to significant improvements in care. We have not assessed the sustainability of this work after the project ended but various governments are currently using their own funds to replicate the use of coaches providing regular support to help facility staff use QI approaches. In addition, we transferred technical know-how to an Indian NGO that is funded by USAID under the MOH mandate to scale up and strengthen the efforts that we made.

### Antenatal care

The results clearly demonstrate that teams can use QI approaches to redesign care delivery processes and improve delivery of antenatal care. Most of these improvements involved reorganizing work stations and processes, coupled with improved recording practices aimed at early identification and management or referral for high-risk conditions. For example, in facilities in Himachal Pradesh, ANC triage was improved through redesign of space and task shifting of staff. Previously, crowds of ANC patients would rush into a hall, where depending upon the patient load and number of staff, they may or may not receive the requisite care. After QI implementation, stations and staff were defined for specific activities, which led to less crowding and fewer missed opportunities. Simultaneously, recording of clinical care practices improved [27] Reporting of hemoglobin measurement and obstetric history taking was done diligently, since these indicators were considered vital for identification of high-risk pregnancy and therefore we see a rapid increase in these processes in the early months of the intervention after which they plateaued. We did not see the same for ANC counseling, exhibited by fluctuating trends, which is considered a routine and mundane job by most staff. It was only after 12 months that counseling showed a gradual improvement; this occurred due to renewed focus on counseling and introduction of guidelines. Our findings are in alignment with another study in Kenya which demonstrated improved adherence to clinical standards of antenatal care, such as measurement of hemoglobin and blood pressure, administration of tetanus toxoid vaccination, and counseling following a QI intervention [28].

### Intrapartum care

We found swift improvement in processes that do not require additional technical skills, are delivered as a single step, and are one-sided (do not involve client compliance issues) such as giving oxytocin within one minute of birth, vitamin K, cord care, and keeping babies dry and warm. For example, improvement could already be seen in oxytocin administration as teams were preparing for the intervention (from 1% in July 2013 to 57% in February 2014). Most facilities were able to apply simple changes like prefilling syringes with oxytocin and disseminating information among staff conducting deliveries to improve administration, and therefore we see a quick increase during the early months of intervention up to May 2014 when it reached 90% and stabilized thereof. Our finding echoes that of another study in Rajasthan where a QI intervention helped increase delivery of oxytocin after birth from 57 to 90% [29]. Early breastfeeding increased over the intervention period (Table 3). The time series chart at the all state aggregate level shows a high reporting in baseline which actually drops once the intervention starts, probably a result of more accurate reporting, picking up again during intervention after which it does not show any increase month to month during intervention period (Fig. 2). Not reaching 90–100% can perhaps be explained by the fact that out of all deliveries, there are some which result in extremely sick newborns or sick mothers who are unable to breastfeed. Keeping babies dry and warm increased to cover almost all babies at project end, which was higher than the reported 80% coverage from the Rajasthan QI study [30]. It could be that our intervention ran for a longer period of time (21 months) during which new steps in improvement had time to become established practices. Facilities were quickly able to raise vitamin K administration to approximately 90%. Achieving the final 10% took longer, as teams had to learn how to give vitamin K to neonates with complications, which required additional care.

### Postpartum care

Although there was increase in the number of vitals check after delivery, not all states demonstrated this increase and hence we did not have statistically significant improvement. Nevertheless, the increase in certain states health staff was achieved by setting a schedule of monitoring vital parameters in the patient’s records and documenting it for all cases. In some facilities, vitals monitoring was improved by reorganization of the labor ward and partnering with family members to inform nurses in case of danger signs [30]. This shows that despite shortage of staff, which is reported as the main limitation in carrying out monitoring of heart rate and blood pressure [3], improvement can be made. Specific changes and strategies associated with improvements in all these elements of care were gathered and are currently available online [31].

Although perinatal mortality showed a decline over time, there is no evidence that the intervention had a significant impact on its decline. This may be because there was an insufficient baseline period or the intervention period was not long enough to affect mortality. Alternatively, the decline we observed could simply be due to secular trends.

### Limitations

There are a number of limitations in our study. The number of facilities varied across the intervention, especially those reporting ANC indicators. Circumstantial reasons such as the departure of a district coach or the implementation of a government program that moved staff to other tasks or achievement in the said improvement goal led to less reporting. Nevertheless, we believe that such factors were not extensive enough to influence the overall trend since the number of facilities was still sufficient in terms of in-patient loading to measure improvement during entire intervention period (Table 1). While data were generally obtained from hospital registers, some data were based on observations of 20 patients or a sample of records. This, however, occurred in the initial stage of the intervention. As the said indicator became an established practice, data from hospital registers became the norm. Furthermore, since we did not have sufficient resources to conduct separate interviews with patients, we do not have their perspective of whether and how clinical care was delivered which may affect our interpretation. Future programmes must include user perspective. Another limitation is that while we analyzed data at the state level, we have not presented individual state results due to limitation in text length. Lastly, we could not estimate the effect of external variables, such as the effect of other interventions to improve care. However, no drastic changes to the macro-level factors (policy and program) occurred in the 26-month period under reporting. We therefore believe that this study supports the effectiveness of QI intervention in improving patient care.

## Conclusion

Lack of quality of care is increasingly being recognized as a major contributing factor to maternal and neonatal mortality in India and other countries. Commonly used strategies to improve the quality of service delivery, including standards and guidelines, training, incentives, and monitoring are necessary but they are not the only strategies available to the health sector. The findings from this study suggest that quality improvement methods, including setting a goal, forming a team, and working iteratively to reach that goal can improve the delivery of facility-based routine clinical practices.

## Additional files

Additional file 1: File name: State data ASSIST. Title of data: Aggregated statewise data of selected indicators. Description of data: Monthly data on all selected indicators across all states providing numerator and denominator of indicators indicating proportion of services conducted. (XLSX 1763 kb)

Additional file 2: File Name: Copy of total records reviewed. Title of data: Aggregated monthly patient records. Description of data: The total number of patient records reviewed per month for broad indicators: total number of ANC records reviewed, total number of deliveries reviewed and total number of newborn records reviewed. (XLSX 9 kb)

## Abbreviations

ANC: Antenatal care
ASSIST: Applying science to strengthen and improve systems
ENC: Essential newborn care
JSY: Janani suraksha yojana
QI: Quality improvement
RMNCH + A: Reproductive, maternal, neonatal, child and adolescent health
SRU: State RMNCHA units
USAID: US agency for international development

## Footnotes

1 Mid-level performance health indicators are the district level indicators (DLHS-3) and moderately performing facilities in terms of quality are selected assuming to improve under QI intervention plan rather than poorly performing and highly performing, since the poorly performing facilities may not be improved only by QI with the existing infrastructure.
